# Who Cares: What Is The Future Landscape Of Geriatric And Gerontology Education

**DOI:** 10.1093/geroni/igaf122.3271

**Published:** 2025-12-31

**Authors:** Isaac Asirifi Boateng, Phyllis Greenberg

**Affiliations:** St. Cloud State University, St. Cloud, Minnesota, United States; St. Cloud State University, St. Cloud, Minnesota, United States

## Abstract

The need for advancements in geriatric and gerontology education has become increasingly vital. The current workforce has not kept pace with aging demographics. There is a low percentage of students choosing geriatrics as a specialty, with fewer than 5% of physicians specializing in this field (Kusmaul, 2023). Approximately 25% of BSW and 20% of MSW students have taken a course in gerontology. Adding to the issue is that nearly 50% of MSW students state that they have little or no interest in working with older adults after graduation (Cummings & Galambos, 2002). This poster examined the current landscape and future directions of medical and social service education regarding older adults. It stresses the importance of a critical look at gaps to equip future providers with the necessary skills and knowledge to address the complex needs of an aging population. This poster seeks to identify significant barriers, such as limited exposure to geriatric and gerontology training, underrepresentation of geriatric specialists, and a lack of funding and incentives for professionals working with an aging population. Proposed recommendations include all future practitioners having a baseline education in working with older adults, advocating for policy reforms to prioritize funding incentives (e.g.; scholarships, assistantships, internships) for students in the field. Overall, addressing these issues and implementing the recommended strategies, gerontology and geriatrics education can better prepare professionals not only to deliver high quality services for older adults but rather connect passion with their WHY of ultimately improving the lives of older adults.

